# High-titer expression of recombinant antibodies by transiently transfected HEK 293-6E cell cultures

**DOI:** 10.1186/1753-6561-9-S9-P40

**Published:** 2015-12-14

**Authors:** Volker Jäger, Johanna Groenewold, Dominik Krüger, Dennis Schwarz, Veith Vollmer

**Affiliations:** 1 Recombinant Protein Expression Group, Helmholtz Centre for Infection Research, 38124 Braunschweig, Germany

## Background

HEK 293-6E cells expressing constitutively a truncated version of EBNA-1 were originally developed at the NRC-BRI in Montreal, Canada. They already proved to be excellent tools for transient transfection and subsequent high-titer production of recombinant proteins[1]. We substantially improved an existing protocol by introducing a number of additional or alternative steps during and after transfection finally resulting in a high-titer production protocol suitable for routine manufacturing of mg to gram quantities of desired proteins.

## Experimental Setup

A recombinant antibody construct introduced into an optimised plasmid vector was used as a model protein for all experiments [2]. A second plasmid for co-expression of an eGFP reporter gene was added at 5%, totalling 1 mg L-1 of plasmid DNA. Cultivation and transfection of HEK 293-6E cells was either done using FreeStyleTM F17 medium (Life Technologies) alone or in combination with SMIF8 2x (Scharfenberg SZS). Optimization experiments were performed in either shaker flasks, bench-top bioreactor systems (which could be operated also in continuous perfusion mode) or the BioLector® 48-well microbioreactor (m2p-labs). Transfection rates were monitored by co-expressed eGFP using a flow cytometer (GuavaEasyCyte) (as well as on-line by fluorescence measurement in the BioLector) and the antibody production by biolayer interferometry (Octet® RED96; Pall fortéBIO). The concentrations of selected metabolites in the supernatant were measured photometrically (GalleryTM, Thermo microgenics).

## Results

Various strategies which have been reported to be beneficial for protein production in other cell lines such as CHO or hybridomas proved to be unsuccessful for HEK 293-6E cells. This includes temperature shifts to either 32 or 34.5 °C (mild hypothermia) [3], moderate (485 mosmol kg-1) or strong (595 mosmol kg-1) increases of the osmolality in the presence of an osmoprotective reagent[4] and the use of either DMSO or lithium acetate [5] in various concentrations for an increased membrane permeablity during transfection. All of these strategies were found to be either negligible or negative on the final yield of the recombinant protein.

Different to that, the histone deacetylaseinhibitors (HDACi) butyrate and valproate were confirmed to be highly beneficial for recombinant protein production withHEK 293-6E cells. Their impact on recombinant antibody production was analysed using a BioLector multi microbioreactor as cultivation system. The success of transient transfection of was monitored on-line by measurement of the fluorescence development in the multiwells as well as offline by taking samples which were made subject to analysis by flow cytrometry. Recombinant antibody accumulation was measured at the end of the experiment seven days post transfection. First of all, it was revealed that reporter gene expression and corresponding measurement methods are neither interchangable nor directly comparable to the expression of the GOI i.e. the recombinant antibody. Antibodies were found at comparable, significantly increased yields using either butyrate or valproate (peaking at 3.75 mM, respectively). No further increase was observed when supplementing both HDAC inhibitors simultaneously.

**Figure 1:**
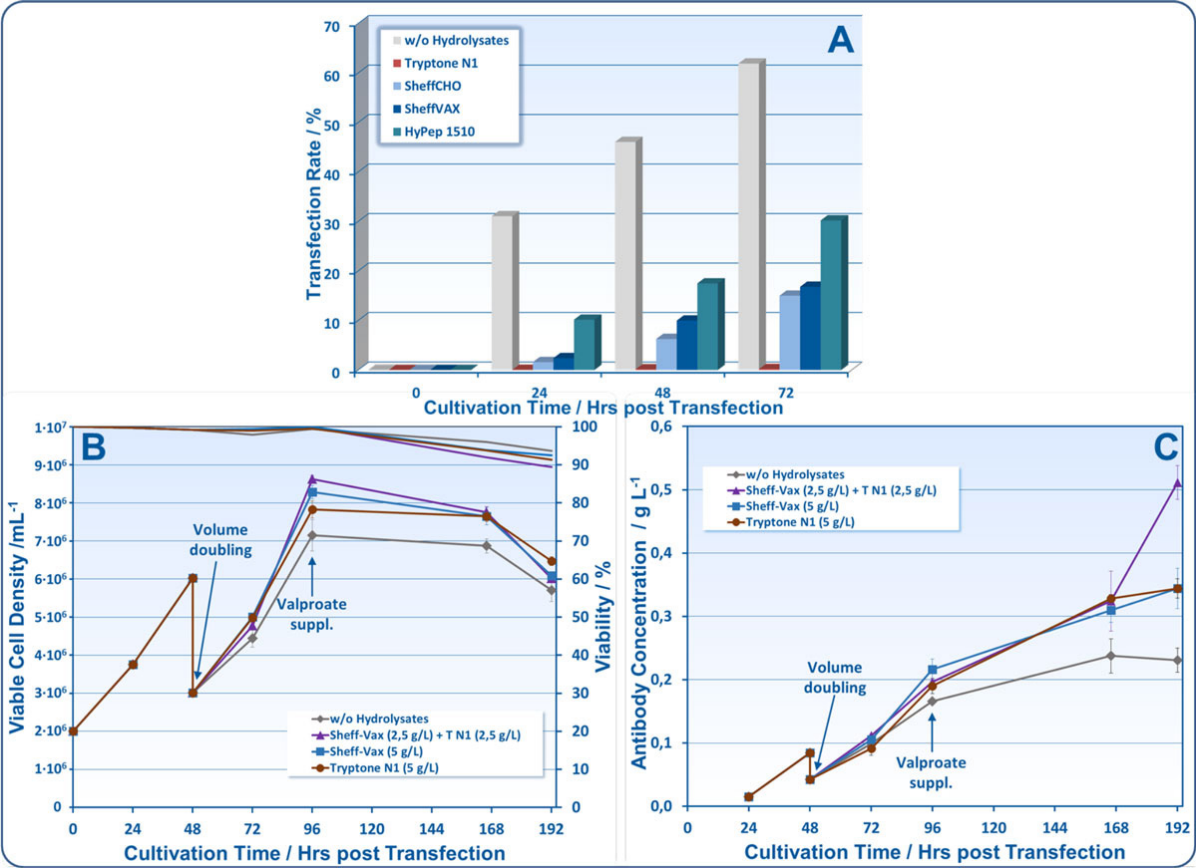
**Influence of protein hydrolysates on the transient transfection process and subsequent recombinant antibody production**. Experiments for expression kinetics were performed in triplicate in 125 mL shake flasks with a final filling volume of 50 mL after doubling 48 hrs post transfection. This was followed by further feeding steps as indicated in Table 1.

All protein hydrolysates tested did completely or drastically inhibit the transfectability of HEK 293-6E cells (Figure 1A). On the other hand, supplementation with protein hydrolysates provided higher cell densities (Figure 1B) and substantially higher recombinant protein concentrations (Figure 1C). The cease of cell proliferation 96 hours post transfection was a result of sodium valproate supplementation. Accordingly, no nutrient limitations or inhibitory accumulations of metabolic byproducts were detected. Tryptone N1, manufactured from casein (Organotechnie), completely inhibited transient transfection of cells but, when supplemented 24 or 48 hrs post transfection at a concentration of 5 g L-1, increased recombinant antibody production. Similar results were obtained using different peptones (HyPep 1510, Sheff-Vax, Sheff-CHO, all from Kerry) with HyPep 1510 showing the lowest inhibitory effect during transfection and Sheff-Vax providing best productivity at 5 g L-1. A further increase in productivity was achieved by blending tryptone N1 with Sheff-Vax (at 2.5 g L-1, respectively) which more than doubled the recombinant protein yield.

Correspondingly, the original transfection and protein production protocol was improved step by step by introducing alternative or additional steps of media supplementation and prolonging the cultivation process. Details of the resulting protocol are listed in Table 1.

**Table 1:** Schedule for transient transfection of HEK 293-6E cells and subsequent feeding

Original transfection protocol			Improved standard transfection protocol	
**- 48 hrs**	Cell seed at 5·10^5 ^ mL^-1 ^*or*	↓		
**- 24 hrs**	Cell seed at 1·10^6 ^mL^-1^		- 24-2 hrs	Cell seed at 1-2·10^6 ^mL^-1^
**0 hrs**	PEI-mediated transfection		0 hrs	Cationicpolymer-mediated transfection
**24-48 hrs**	Tryptone feed (0.5% TN1)			
**48 hrs**	Harvest of intracellular proteins *or*		48 hrs	Hydrolysates (0.5%) + volume doubling
			72 hrs	Glucose feed (4.5 g L^-1^)
			96 hrs	Sodium valproate feed (3.75 mmol L^-1^)
**120 hrs**	Harvest of extracellular proteins			
			144 hrs	*or*
			168 hrs	*or*
			192 hrs	Harvest of extracellular proteins
**Antibody yields in the range of 130 to 150 mg L^-1^**			**Antibody yields in the range of 500 to 800 mg L^-1^**	

## Conclusions

Similar to the effect on many other cell lines both sodium butyrate and valproate substantially increase recombinant protein productivity of HEK 293-6E cells. However, cells do also respond with a reduced growth and finally a decline of viability which suggests a careful adjustment of both the concentration and the moment for HDACi supplementation. Combinations of both reagents did not reveal any cummulative effect. Thus, valproate was selected in order to minimise costs. Protein hydrolysates severely interfere with the process of cationic polymer-mediated transient transfection. However, when supplemented at a later stage, selected formulations ensure improved cell-specific recombinant protein productivity combined with a higher volumetric yield. On the contrary, many successful strategies reported for the cultivation of CHO cells proved to be unsuitable for HEK 293-6E. In addition, this cell line is also behaving quite different compared to other HEK 293 cell lines such as HEK 293 EBNA (formerly available from Life Technologies). By applying an improved transfection protocol the resulting final concentrations of recombinant antibodies could be increased by a factor of 4 to 5 and the yield based on the amount of used plasmid DNA by a factor 8 to 10.
